# Effects of a mindfulness-based interventions on stress, burnout in nurses: a systematic review and meta-analysis

**DOI:** 10.3389/fpsyt.2023.1218340

**Published:** 2023-08-03

**Authors:** Qi Wang, Fang Wang, Shurong Zhang, Chaofan Liu, Yue Feng, Junzhu Chen

**Affiliations:** ^1^School of Nursing, Chengdu University of Traditional Chinese Medicine, Chengdu, Sichuan, China; ^2^Guang’an Hospital of Traditional Chinese Medicine, Guang’an, Sichuan, China

**Keywords:** mindfulness, nurse, burnout, pressure, systematic review

## Abstract

**Background:**

Stress in the healthcare environment causes negative effects in nurses such as burnout, anxiety, and depression. The COVID-19 pandemic has resulted in increased pressure on medical staff globally, highlighting the potential benefits of mindfulness-based interventions in reducing nurses’ stress levels. Despite numerous studies exploring the effect of mindfulness-based training on nurses, the results remain inconclusive.

**Objective:**

To systematically evaluate the impact of mindfulness training on nurse’s performance and increase the certainty of existing evidence.

**Methods:**

This study searched various databases, including EBSCO, Embase, Web of Science, PubMed, ProQuest, Scopus, Cochrane Online Library, Wanfang, SinoMed, CNKI, and VIP, for randomized controlled trials on the impact of mindfulness-based interventions for nurses up until 02 December 2022. Two investigators independently screened and extracted data from the articles, while also assessing the risk of bias. The data was analyzed using RevMan 5.4 software.

**Results:**

This review identified 15 studies out of the 2,171 records retrieved, consisting of a total of 1,165 participants who were randomized. Post-intervention analysis provided very-low certainty evidence of moderate effectiveness of mindfulness-based training in reducing stress [standardized mean difference (SMD) = −0.81; 95% confidence interval (CI) = −1.11 to −0.52], with no significant effect on anxiety (SMD = −0.30; 95% CI = −0.72 to 0.13) or depression (SMD = −0.24; 95% CI = −0.55 to 0.07). However, the training was effective in reducing burnout, as demonstrated by the lower scores for emotional exhaustion (SMD = −4.27; 95% CI = −5.94 to −2.59) and depersonalization (SMD = −2.89; 95% CI = −4.24 to −1.54) and higher scores for personal accomplishment (SMD = 2.81; 95% CI = 0.12 to 5.50). There was a sustained improvement in stress levels in the short-term (≤3 months), with delayed benefits for burnout. However, only two studies were available for later follow-ups, and there was no significant evidence of long-term effects.

**Conclusion:**

Mindfulness-based training may be a viable intervention for improving the psychological wellbeing of nurses, including reducing stress, burnout.

**Systematic review registration:**

https://www.crd.york.ac.uk/PROSPERO/, identifier CRD42023387081.

## 1. Introduction

The healthcare industry can have negative impacts on the overall health and wellbeing of healthcare professionals due to numerous factors, including shift work, stress, caregiving responsibilities, colleague relationships, and performance targets. These factors can result in feelings of anxiety, depression, stress, burnout, and fatigue among healthcare professionals (1). The recent COVID-19 pandemic has further exacerbated the negative effects on healthcare providers worldwide. A cross-sectional study conducted found that nurses who worked in intensive care units, COVID-19-designated hospitals, and departments treating COVID-19 patients had significantly higher scores related to mental health outcomes when compared to other healthcare professionals (2).

Burnout is a critical consequence of long-term exposure to work-related stressors and can lead to emotional exhaustion, depersonalization, and a lack of personal fulfillment (3). This condition can also result in negative emotions, substance abuse, and suicidal ideation (4). When it comes to physicians, burnout may cause longer recovery times, medical errors, decreased productivity, and increased turnover rates (5). The recent COVID-19 pandemic has had a significant impact on the burnout rates of healthcare workers (6). Research conducted in Turkey has consistently demonstrated that nurses unfortunately experience higher rates of burnout syndrome than other healthcare professionals, such as physicians and medical technicians (7). Occupational stress is a psychophysical reaction that can occur when an individual’s job demands exceed their abilities or resources, or when their needs are excessively met (8). This physical and emotional condition can have negative effects, especially if employees do not have access to the resources, abilities, and needs required for their work (9). Nurses who experience long-term exposure to job-related stressors and burnout are at risk of significant physical and mental health problems, such as endocrine disruption, insomnia, weakened immune systems, heart disease, diabetes, and obesity, all of which can negatively impact the quality of their nursing care (10). Therefore, addressing burnout has become a crucial public health policy priority globally, with healthcare institutions encouraged to prioritize investments in the overall wellbeing of nurses, particularly those who are new to their careers (11).

Charzyńnska’s research outlines two major types of spiritual coping: positive and negative (12). Positive spiritual coping typically involves cognitive and behavioral strategies that are employed to address challenging situations. Meditation is a widely recognized positive form of spiritual coping with many benefits, including improved self-esteem, mindfulness, overall wellbeing, and reduced stress and blood pressure (13). It has also been shown to be particularly helpful in managing job-related stress. Additionally, Chow et al. (14) found that healthcare providers who work with COVID-19 patients can benefit from positive religious coping as a way of reducing stress, anxiety, and depression. Mindfulness-based interventions (MBIs) are a proven and effective form of psychotherapy for addressing various mental health disorders (15, 16). At its core, mindfulness involves self-regulating one’s attention and focusing on the present moment with curiosity, receptivity, and openness (17–19). Neuroimaging studies also support the positive effects of mindfulness on the brain and behavior and reveal brain changes in response to mental training over the long-term, particularly in the self-specifying and self-relational thinking networks (20). The long-term practice of mindfulness can lead to improvements in individual and social wellbeing (20).

Systematic reviews on the effect of mindfulness-based interventions (MBIs) on the mental health outcomes of nurses have presented contradictory findings, especially in relation to anxiety and depression (21–23). These inconsistencies could arise from differences in participant characteristics, intervention techniques, and assessment measures, leading to diminished reliability of these studies, and making it difficult to establish a clear research direction. Systematic reviews and meta-analyses that rely on these studies may introduce bias and compromise recommendations for medical professionals, thereby impacting the physical and mental wellbeing of healthcare workers. Additionally, previous systematic reviews suffer from limited sample sizes and inadequate differentiation between short and long-term effects of interventions, resulting in unpredictable and untrustworthy results (24). Hence, this study intends to conduct a comprehensive systematic review and meta-analysis of MBIs for nurses to offer evidence-based recommendations for clinical practice.

## 2. Methods

This review was conducted following the PRISMA guidelines and registered on PROSPERO (registration number: CRD42023387081). As a review with meta-analysis, no ethical approval was necessary.

### 2.1. Search strategy

A comprehensive study was conducted using various databases, namely, PubMed, Embase, EBSCO, Web of Science, ProQuest, Scopus, and Cochrane Online Library. Furthermore, four Chinese databases were also included in the analysis, namely, National Knowledge Infrastructure (CNKI), Chinese Biomedical Literatures database (SinoMed), Wanfang Digital Periodicals (WANFANG), and Chinese Science and Technology Periodicals (VIP). Data was collected from the inception of these databases until December 2022. Key terms, including “nurse/nurse*/Nursing Personnel/Registered Nurses/Nursing Staffs” AND “mindfulness/mindful*/Mindfulness-based/mindfulness meditation/mindfulness inter-vention/Mindfulness-based therapies/mindfulness training/Mindfulness-based stress reduction/mindfulness-based cognitive” AND “randomized controlled trial/randomized/placebo” were used. The search strategy outlined above was utilized in all of the previously mentioned databases. No restrictions regarding publication status, date, or year were imposed, but only studies published in English or Chinese were considered. Additionally, the reference lists of identified articles were manually searched for relevant studies. Other studies were accessed via Baidu Xueshu and Google Scholar. Retrieval outcomes were managed and stored using Endnote software. Supplementary Table 1 illustrates the PubMed search strategy.

### 2.2. Inclusion and exclusion criteria

This study selected: (a) randomized controlled trials (RCTs); (b) Registered nurses, aged ≥18 years, working in a clinical or outpatient setting; (c) focus on mindfulness-based interventions; and (d) The primary outcomes assessed were stress and burnout, while secondary outcomes included negative emotions such as depression and anxiety. Exclusion criteria included studies not in English or Chinese language, animal studies, meta-analyses, reviews, case reports, conference abstracts, duplicate studies, and studies with incomplete or unusable data.

### 2.3. Date extraction

The authors applied inclusion criteria to the titles and abstracts. In cases of disagreement, two reviewers (WQ and ZSR) discussed the matter, or a third researcher (FY) was consulted. Abstracts deemed possibly or extremely relevant were obtained in full text and assessed by two reviewers (WQ and ZSR) for eligibility. Discrepancies in study inclusion were resolved through consensus. After data retrieval, two reviewers (WQ and ZSR) checked for accuracy and assessed study quality. The extracted data included information on the first author, country, publication year, sample size, age, intervention, training intensity, measures time point, and outcomes.

### 2.4. Risk of bias

We used utilized the Cochrane risk of bias assessment tool (25) to evaluate the included studies for potential bias. Seven domains of bias were evaluated on a three-point rating scale (high, low, or unclear risk of bias), with ratings of A, B, or C reflecting high, moderate, or low probability of bias, respectively. Studies rated as Grade C were omitted from the analysis. Two reviewers (WQ and ZSR) independently assessed risk of bias, with discrepancies resolved by discussion or a third reviewer (FY). The results of the evaluation are presented in graphs generated using Review Manager 5.4.

### 2.5. Assessment of the quality of the evidence–GRADE

The research utilized the GRADE system to evaluate the validity of the data evidence. The results of studies with large and low quality provide evidence that interventions produced positive outcomes. Supplementary Table 2 contains additional details on the evidence evaluation.

### 2.6. Statistical analysis

Data analysis and pooled scores calculation were performed using RevMan 5.4 software. For continuous variables, mean difference (MD) and standardized mean difference (SMD) were used as summary measures for the same outcome measured by same or different scales, respectively. Statistical heterogeneity of the studies included was determined through the *Q* test (*P*-value) and *I*^2^. If *P* ≤ 0.1, or *I*^2^ ≥ 50%, there was statistical heterogeneity between the studies, and a random effects model was applied. Otherwise, a fixed effects model was used for the meta-analysis. Narrative descriptions were used to resolve heterogeneity that could not be combined. Sensitivity analysis through elimination of included studies was conducted to examine the stability of the results. stata16 software was used to generate the sensitivity analysis chart and Egger test was used to detect publication bias (when *P <* 0.05, publication bias was significant).

## 3. Results

### 3.1. Literature search results

Initially, 2,190 records were retrieved. From these, 749 duplicate records were excluded, and 1,327 records were further filtered by title and abstract. Among these, 99 studies did not meet the criteria for inclusion after being fully reviewed, leaving 15 studies. A flow diagram of the article screening process according to PRISMA is shown in Supplementary Figure 1 and the PRISMA checklist is shown in Supplementary Table 3.

### 3.2. Study characteristics

Supplementary Table 4 outlines the Characteristics of the included studies in this analysis. The review encompassed a total of 15 studies, comprising 1,165 nurses. The publications were varied, ranging from 2015 to 2022. The sample sizes for the studies varied from 40 to 106 participants. Regarding these studies, nine (26–34) were from China, two (35, 36) were from the United States, four were from Japan (37), Iran (38), Portugal (39), and Turkey (40). The average age ranged from 21 to 60. Among all randomized controlled trials included, nine studies (27–30, 32–34, 38, 39) used mindfulness-based interventions, two studies (31, 37) used mindfulness-based stress reduction interventions, two studies (36, 40) used mindfulness breathing therapy (one of which included music therapy), one study (26) used mindfulness coloring, and one study (35) used yoga. The duration of 11 studies (27–36, 38) was 8 weeks, one study (39) lasted for 6 weeks, one study (37) had a longer duration of 52 weeks, and the specific duration of two studies (26, 40) was not mentioned. The intervention intensity was mostly once a week, with 8 sessions per week. The intervention length ranged from 5 days to 52 weeks, while each individual session lasted anywhere from 10 min to 3 h. Six studies (27, 29, 31, 34, 37, 38) involved follow-up after intervention, ranged from 1 to 6 months.

### 3.3. Risk of bias assessment results

Of the 15 included RCTs, One study (35) only mentioned randomization but did not describe the specific randomization method; four studies (31–33, 39) did not describe the hidden allocation scheme specifically; five studies (26, 29, 31, 36, 40) attempted to use blinding and online data collection by intervention personnel, due to the impact of intervention measures, blinding could not be implemented on the research subjects. However, the outcome indicators were all objective, so the effect of not implementing blinding on the results was not significant; 12 articles (26, 27, 31–40) implemented blinding for outcome assessors; all 15 articles (26–40) clarified the inclusion criteria for the research subjects, and comparability was maintained among groups; no incomplete data report was found (Supplementary Figure 2). The methodological quality evaluation was all grade B, and the literature quality was acceptable (Supplementary Table 5).

### 3.4. Meta-analysis

#### 3.4.1. Stress

In terms of stress reduction, large effect sizes were observed both immediately post-intervention (SMD = −0.81, *P* < 0.01, 95% CI: −1.11, −0.52) and at the 3-month follow-up (SMD = −0.69, *P* < 0.01, 95% CI: −1.08, −0.31), with the latter effect size being moderate in size. Furthermore, a single study (31) suggested that there was a large effect size for stress training at long-term follow-up (SMD = −0.86, *P* < 0.01, 95% CI: −1.12, −0.60).

#### 3.4.2. Burnout

The *I*^2^ results indicated moderate heterogeneity across the studies, with values of 39, 55, and 73% for emotional exhaustion (EE), depersonalization (D), and personal accomplishment (PA), respectively. For EE, there was a difference in means between the intervention and control groups, with a lower mean value observed in the intervention group (mean difference = −4.27, *P* < 0.01, 95% CI: −5.94, −2.59). Similarly, for D, the intervention group had a lower mean value (mean difference = −2.89, *P* < 0.01, 95% CI: −4.24, −1.54). For PA, a difference was also observed in favor of the intervention group (mean difference = 2.81, *P* = 0.04, 95% CI: 0.12, 5.50). However, no significant effect of mindfulness-based interventions (MBIs) on any dimension of burnout was found at long-term follow-up.

#### 3.4.3. Other outcomes

At post-intervention, the results did not indicate any significant effect of MBIs on anxiety (SMD = −0.30, *P* = 0.17, 95% CI: −0.72, −0.13) or depressive symptoms (SMD = −0.24, *P* = 0.13, 95% CI: −0.55, −0.07). The respective forest plots for these outcomes are provided in Supplementary Figure 3–6.

### 3.5. Subgroup analysis

Subgroup analysis was conducted in this study to investigate the heterogeneity of stress based on country, outcome measures, and intervention duration. Supplementary Figures 7A–C were used for this analysis. However, it was not possible to conduct subgroup analysis for burnout due to the limited number of studies (<10). The authors did not assess the risk of bias due to missing results based on funnel plot asymmetry, in accordance with the recommendations in the Cochrane Handbook (41).

#### 3.5.1. Regarding the country of study

The study included six studies from China and four studies from other countries. In the subgroup analysis based on country, the results showed that the studies from other countries were less heterogeneous than those from China. The SMD was −0.92, *P* < 0.01, 95% CI: −1.34, −0.52, and *I*^2^ was 79% for the China subgroup. For the other country subgroup, SMD was −0.62, *P* < 0.01, 95% CI: −0.96, −0.28, and *I*^2^ was 47%. Both subgroups had a moderately positive effect on improving stress.

#### 3.5.2. Regarding the outcome measures of study

The outcome measure was PSS in 5 studies and other in 5 studies. In the former subgroup: MD = −4.14, *P* < 0.01, 95% CI: −5.26, −3.02; *I*^2^ = 0%, and in the latter subgroup: MD = −6.82, *P* < 0.01, 95% CI: −11.40, −2.23; *I*^2^ = 95%. The findings indicated that the studies exhibited no significant variation with regards to the outcome measure of perceived stress scale (PSS). Moreover, both groups displayed a enhancement in the mitigation of stress levels.

#### 3.5.3. Regarding the intervention duration of study

The length of the intervention was classified as short-term if it was less than 8 weeks and long-term if it was 8 weeks or longer. Out of the total number of studies, two investigated the effectiveness of short-term interventions, while seven assessed the impact of long-term interventions. In one study (40), the intervention’s duration was not specified. The results indicated that both short-term (SMD = −0.42, *P* < 0.01, 95% CI: −0.72, −0.11; *I*^2^ = 0%) and long-term interventions (SMD = −0.99, *P* < 0.01, 95% CI: −1.32, −0.63; *I*^2^ = 72%) had distinct positive effects on stress reduction, with the long-term interventions resulting in a more substantial improvement.

### 3.6. Sensitivity analysis and publication bias

A sensitivity analysis was conducted to assess stress, and the findings indicated that the estimated value was similar to the total combined value, which suggests that the results of the meta-analysis are dependable and consistent as shown in Supplementary Figure 8A. Sensitivity analysis plots for other results are shown in Supplementary Figures 8B, C. Furthermore, the Egger test was utilized to evaluate the potential for publication bias, yielding a score of (*Z* = 0.318), indicating a low risk of publication bias in this research.

## 4. Discussion

Mindfulness-Based Interventions (MBIs) have numerous benefits for individuals, including increased recognition of current emotions and improved focus on present tasks, which can promote inner peace and happiness (42). Previous research has indicated that MBIs are effective in promoting better mental health for individuals. Specifically, studies have shown that MBIs are effective in reducing symptoms of common psychological issues such as depression, anxiety, and stress, while simultaneously promoting overall psychological wellbeing. (43–46). Our review stands out from previous ones in the field due to its unique focus on nurses and its inclusion of more comprehensive and experimental studies. Moreover, we have taken a comprehensive approach by including all relevant studies featuring interventions based on mindfulness, meditation, and relaxation techniques, which may have significant implications for evaluating their effects on nurses, separate from the effects of mindfulness alone. While a systematic review (17) has examined psychological distress among nurses, it only included English publications released from 2011 to 2021. Suleiman–Martos’ review (24) included mindfulness training in their intervention and sampled nurses, but the only outcome examined was burnout. Other systematic reviews have delved into the effectiveness of mindfulness among other healthcare professionals (47, 48), whereas our review focuses specifically on nurses and is, to the best of our knowledge, the only one to do so.

This review contributes to the existing literature on the effectiveness of MBIs in improving the wellbeing of nurses. We identified 15 Randomized Controlled Trials (RCTs) conducted in clinical settings from inception to December 2022, and conducted pairwise meta-analyses across four outcomes. Our findings indicated moderate to large positive effects of MBIs on perceived stress and burnout immediately post-intervention and in the short-term, but limited evidence for training effects on anxiety and depressive symptoms. The review also highlighted the need for further research on the long-term effects of MBIs on nurses, as the evidence on this outcome was based on only a few studies. However, it is important to note that the certainty of evidence was low and further research is needed to strengthen the conclusions. It is also essential to consider the limitations of this study, such as the small number of studies with long-term follow-up assessments. Future reviews should address these limitations and provide more conclusive evidence on the effectiveness of MBIs in improving nurses’ wellbeing.

Nurses face a range of stressors in the healthcare field that can greatly affect their overall health and wellbeing. Such stressors may include working extended hours, managing patients’ pain, dealing with loss and emotional distress, providing end-of-life care, and supporting the families of patients with critical illnesses (23). Recent research has indicated that the inherent stress found within healthcare professions can result in a variety of negative outcomes, including lowered job satisfaction, depression, interpersonal discord, and psychological distress (49). These stressors can result in increased risk of harm to patients. During the pandemic, nurses face additional stressors beyond their typical job-related pressures. These include psychological burdens resulting from disease-related adversity (such as increased risk of infection, pain, and mortality) and restrictive containment measures (such as isolation) (21, 50). Findings from a recent study suggest that MBIs represent a useful tool for reducing perceived stress among nurses. This may be due to the fact that mindfulness enhances one’s awareness of the present moment, creating a clearer and more accurate perception and encouraging a correct reevaluation of stressors (51). Hölzel et al. (52) have indicated that mindfulness based stress reduction can considerably enhance activation in and connectivity between various brain regions that are critical for successful emotion regulation (53). In terms of mechanisms of change associated with mindfulness-based stress reduction (MBSR), self-compassion and mindfulness have emerged as crucial mediators for reducing stress (54). Mindfulness facilitates the prevention of habituation and the constant assumptions associated with mindlessness (20). Additionally, a possible association between mindfulness and psychological wellbeing has been hypothesized as being mediated by relaxation, meta-cognitive insight, contact with reality, and exposure (55). Follow-up assessment outcomes revealed substantial reductions in perceived stress among nurses immediately after intervention as well as at the 3-month follow-up test, consistent with previous studies (10, 49). Only one study (31) has investigated sustained stress beyond 3 months, with sustained stress being demonstrated. However, due to the small number of studies, the evidence remains inconclusive and warrants further research.

Occupational burnout is a state of being where an individual experiences a decline in their sense of self, a feeling of overwhelming emotional exhaustion, and a diminished sense of personal achievement. Mental health professionals in various countries, including Singapore, have expressed concern about this syndrome, as it can adversely affect nursing quality and staff turnover rates (56). In one study, it was found that nurses who reported high levels of burnout were significantly more likely to intend to leave their current job (43%) compared to non-burned-out nurses (11%) within the next year (23). Additionally, another study demonstrated a significant correlation between the number of patients assigned to a nurse and their likelihood of experiencing emotional exhaustion and burnout. Specifically, for every additional patient assigned to a nurse, their risk of burnout increased by 23%. The result indicated that for each added patient assigned to a nurse, there was a 23% increase in the likelihood of experiencing burnout (57). Several studies (10, 23, 24) have reported that mindfulness training is an effective method for reducing burnout levels. Reduced levels of emotional exhaustion and depersonalization can be observed, along with enhanced personal achievement, as a potential outcome. The findings of this study align with these previous research studies. There was high heterogeneity between studies, and after removing Watanabe’s study (37), the heterogeneity significantly decreased, possibly due to the different duration, intensity or location of intervention, indicating low certainty evidence. According to some authors (58, 59), the duration of the benefits obtained from interventions did not show significant changes or even had an increase in post-intervention burnout scores. One possible explanation for this outcome is the lack of adherence by the nurses who participated in the studies. Consistent training has been shown to be effective in maintaining low levels of burnout. Regular mindfulness interventions targeting emotional exhaustion in nurses may help alleviate burnout and improve nursing quality.

Research has established that elevated levels of job stress are associated with a greater vulnerability to mood and anxiety disorders (42). Healthcare teams reported significantly higher anxiety and depression scores during the COVID-19 pandemic (60). Mindfulness training was found to have a positive impact on nurses’ cognitive abilities (61). Brain imaging studies have indicated that mindfulness training leads to enhanced brain activity, which is correlated with positive emotions and improved emotional regulation (62, 63). Moreover, some researchers have noted an improvement in individuals’ reactions to traumatic situations (64), as well as a reduction in salivary cortisol levels- a stress biomarker- following mindfulness training (65). The studies included in this meta-analysis (28–30, 32, 36–38) reported a reduction in difficulty regulating emotions and lower scores in negative emotions such as anxiety and depression after mindfulness intervention. Nevertheless, the analysis of multiple studies did not indicate any notable effects of MBIs on symptoms related to depression or anxiety, and no significant relief of symptoms or other outcomes were observed. These findings are in accordance with the conclusions drawn from a comprehensive systematic review. (21). The absence of evidence in this study regarding mindfulness having a significant impact on anxiety and depression symptoms should not be perceived as training having negligible effects on these outcomes in nurses. Since there are limited long-term follow-up studies, the scarcity of evidence beyond 3 months after the intervention could be the reason. Therefore, any conclusions from this study should be viewed as preliminary and subject to revision based on future reviews, which provide more robust evidence to better understand the stability of mindfulness intervention effects.

The implementation region of MBIs was subdivided into China and other countries for subgroup analysis. The outcomes indicated that the heterogeneity of studies in other countries reduced (*I*^2^ = 47%), suggesting that the country could be one of the sources of heterogeneity. Conversely, the heterogeneity of studies in China increased (*I*^2^ = 79%), which may be linked to the specific implementation and variances in the mindfulness interventions scales. Besides, subgroup analysis was performed on outcome measures and intervention duration. The PSS group and the short-term intervention group’s heterogeneity decreased significantly. However, the short-term intervention group only comprised two studies, and diverse scales may be the primary source of heterogeneity. Thus, using internationally recognized and standardized scales for large-magnitude multicenter studies is recommended to provide more authentic and dependable evidence. Long-term mindfulness-based interventions (≥8 weeks) have a better positive impact on stress than short-term intervention (<8 weeks). This finding indicates that the effectiveness of MBIs on stress reduction is associated with the length of the intervention, which is consistent with previous findings by Xie et al. (27), where the positive effect was only observed in longer interventions. Establishing the impact of mindfulness-based interventions on stress requires adequate course settings, including appropriate training content and program duration (42). Although our study demonstrated favorable effects of MBIs on nurse stress, variations in training content, duration, and frequency among the included studies precluded definitive conclusions about the preferred mindfulness-based intervention course settings. Therefore, more research is needed to explore this topic further.

## 5. Limitations

First, Some of the studies analyzed in this meta-analysis may have methodological limitations, including challenges in implementing blinding of the study participants and interveners during the intervention, as well as incomplete reporting of blinding. The stability and reliability of the research conclusions may be impacted by these identified limitations. Additionally, the review examined a broad range of eligible trials, however, the overall quality of the collected data was limited. We do not deny the possibility that these studies have incomplete data and other biases, and even some studies that do not provide the mean and/or standard deviation are excluded. Among all the included studies, the basic treatments of each study were different, and some outcome indicators used self-rating scales as subjective evaluation indicators, which may affect the reliability of the research results. Therefore, the evidence presented in this study may have limitations. Additionally, the search was confined to publicly published Chinese and English literature, which may have created publication bias by omitting some relevant studies. Furthermore, although an effort was made to consider the impact of different follow-up periods on outcome indicators, the time span ranged from 5 days to 52 weeks. There are few long-term follow-up studies of more than 3 months, resulting in lower certainty of the evidence.

## 6. Conclusion

In conclusion, this systematic review with meta-analyses shows that mindfulness-based interventions can effectively reduce stress and fatigue among nurses. However, there is insufficient evidence to suggest that MBIs can effectively address anxiety and depression in this group. It is necessary to conduct more high-quality research to identify the most effective intervention components, with larger sample sizes, longer follow-up periods, and diverse study designs. To maintain the physical and mental health of nursing staff, and improve their emotional regulation abilities and psychological states, nursing managers can schedule regular MBIs for them. This will not only enhance their ability to provide high-quality nursing care, but also promote their overall wellbeing. In addition, nursing staff face similar organizational stressors as other healthcare professionals, such as physicians. Therefore, the lessons learned from resilience training interventions for nurses may also be applicable to other groups.

## Data availability statement

The original contributions presented in this study are included in this article/Supplementary material, further inquiries can be directed to the corresponding author.

## Author contributions

QW, SZ, and FW conceived and designed the study. QW and YF were responsible for conducting the search, acquiring and interpreting the data, and drafting the manuscript. JC performed the meta-analysis and generated the figures. CL played a role in developing the study concept and drafting the tables. QW, CL, and YF were responsible for study design and data verification. FW reviewed and revised the manuscript, as well as provided funding for the study. The final version of the manuscript was comprehensively reviewed by all coauthors with complete access to study data. Together, they came to a mutual decision to submit the manuscript for publication.
