# Supplementary material for: Effects of a mindfulness-based interventions on stress, burnout in nurses: a systematic review and meta-analysis
Source: Front Psychiatry. 2023 Aug 3;14:1218340. doi: 10.3389/fpsyt.2023.1218340 (PMC10434780; doi:10.3389/fpsyt.2023.1218340)
Supplement: Supplementary file 1 [file Data_Sheet_1.docx]

Supplementary Material

**Effects of a mindfulness-based intervention on stress, burnout in nurses: A systematic review**

Fang Wang *, Qi Wang, Shurong Zhang, Chaofan Liu, Yue Feng, Junzhu Chen

*** Correspondence:** Fang Wang:wangf7640@163.com

# Supplementary Figures and Tables

## Supplementary Tables

**Supplementary table 1. The search strategy of the PubMed database**

|  |  |
| --- | --- |
| #1 | Mindfulness [mh] |
| #2 | Mindfulness [tiab] OR Mindful* [tiab] OR Mindfulness-based [tiab] OR Mindfulness meditation [tiab] OR Mindfulness intervention [tiab] OR Mindfulness-based intervention [tiab] OR Mindfulness- based therapies [tiab] OR Mindfulness training [tiab] OR Mindfulness decompression therapy [tiab] OR Mindfulness-based stress reduction [tiab] OR Mindfulness-based cognitive [tiab] OR MBCT [tiab] OR MBSR [tiab] |
| #3 | #1 OR #2 |
| #4 | Nurse [tiab] OR Nurs* [tiab] OR Nursing Personnel [tiab] OR Registered Nurses [tiab] OR Nursing Staffs [tiab] OR Licensed Practical Nurse [tiab] OR Licensed Vocational Nurses [tiab] |
| #5 | Nurses [mh] |
| #6 | #4 OR #5 |
| #7 | Randomized controlled trial[pt] |
| #8 | Randomized controlled trial [tiab] OR Randomized [tiab] OR Placebo [tiab] OR RCT [tiab] |
| #9 | #7 OR #8 |
| #10 | #3 AND #6 AND #9 |
| Note: mh: MeSH; tiab: tittle/abstract; pt: publication type | |

**Supplementary Table 2. Certainty of evidence rating (GRADE)**

| **Stress interventions compared to control condition for nursing staff** | | | | | | |
| --- | --- | --- | --- | --- | --- | --- |
| **Patient or population:** registered nurses working in clinical or outpatient settings, from various specialties and medical departments, aged 18 years and older, independent of gender and health status  **Setting:** any healthcare sectors (e.g., psychiatric departments, intensive care unit, surgery, oncology)  **Intervention:** any mindfulness-based interventions, irrespective of content, duration, setting or delivery mode  **Comparison:** no intervention, wait-list control, treatment as usual (TAU) | | | | | | |
| **Outcomes** | **Anticipated absolute effects* (95% CI)** | | **Relative effect (95% CI)** | **No. of participants (studies)** | **Certainty of the evidence (GRADE)** | **Comments** |
|  | **Risk with control** | **Risk with resilience interventions** |  |  |  |  |
| **Stress symptoms or perceived stress**  **Measured by:** investigators measured stress symptoms or perceived stress using different instruments; lower scores mean lower stress symptoms or perceived stress  **Timing of outcome assessment:** post-intervention | See comment | The mean stress or stress perception score in the intervention groups was, on average, **0.81 standard deviations lower** (1.11 lower to 0.52 higher) | - | 763  (10 RCTs) | ⊕⊕⊕⊝  **moderate^a^** | - |
| **Burnout symptoms**  **Measured by:** investigators measured burnout symptoms using consistent instruments; lower scores mean lower burnout symptoms  **Timing of outcome assessment:** post-intervention | See comment | The mean emotional exhaustion score in the intervention groups was, on average, **4.27 standard deviations lower** (5.94 lower to 2.59 higher);  The mean depersonalization score in the intervention groups was, on average, **2.89 standard deviations lower** (4.24 lower to 1.54 higher);  The mean personal accomplishment score in the intervention groups was, on average, **2.81 standard deviations lower** (0.12 lower to 5.50 higher); | - | 404  (5 RCTs) | ⊕⊕⊝⊝  **Low^b^** | - |
| **Anxiety symptoms**  **Measured by:** investigators measured anxiety symptoms using  different instruments; lower scores mean lower anxiety symptoms  **Timing of outcome assessment:** post-intervention | See comment | The mean anxiety score in the intervention groups was, on average, **0.35 standard deviations lower** (0.69 lower to 0.02 higher) | - | 398  (5 RCTs) | ⊕⊝⊝⊝  **Very low^c^** | - |
| **Depressive symptoms**  **Measured by:** investigators measured depressive symptoms using different instruments; lower scores mean lower depressive symptoms  **Timing of outcome assessment:** post-intervention | See comment | The mean depression score in the intervention groups was, on average, **0.24 standard deviations higher** (0.47 lower to 0.01 higher) | - | 294  (4 RCTs) | ⊕⊝⊝⊝  **Very low^d^** | - |
| ***The risk in the intervention group** (and its 95% CI) is based on the assumed risk in the comparison group and the **relative effect** of the intervention (and its 95% CI). | | | | | | |
| **GRADE Working Group grades of evidence**  **High certainty:** we are very confident that the true effect lies close to that of the estimate of the effect  **Moderate certainty:** we are moderately confident in the effect estimate; the true effect is likely to be close to the estimate of the effect, but there is a possibility that it is substantially different  **Low certainty:** our confidence in the effect estimate is limited; the true effect may be substantially different from the estimate of the effect  **Very low certainty:** we have very little confidence in the effect estimate; the true effect is likely to be substantially different from the estimate of effect | | | | | | |
| Note. CI: Confidence interval; RCT: Randomized controlled trial; SD: standard deviation; SMD: standardized mean difference.  ^a^Downgraded two levels due to study limitations (mostly unclear risk of selection bias, partly high risk of attrition bias, high risk of performance and detection bias), and one level due to indirectness (studies limited to certain participants [young and middle-aged adults], interventions [e.g., group and combined setting, face-to-face and combined delivery, moderate and high intensity, mindfulness-based training and combination] and comparators [no intervention, wait-list]).  ^b^Downgraded two levels due to study limitations (unclear risk of selection bias, partly high risk of attrition bias, mostly high risk of performance and detection bias), and one level due to indirectness (studies limited to certain interventions [face-to-face delivery, combined theoretical foundation, moderate and high intensity]), and two levels due to imprecision (< 400 participants; 95% CI wide and inconsistent).  ^c^Downgraded two levels due to study limitations (unclear or partly high risk of selection bias, mostly high risk of attrition bias, mostly high risk of performance and detection bias), one level due to indirectness (studies limited to certain participants [young and middle-aged adults], interventions [e.g., group and combined setting, face-to-face and combined delivery, moderate and high intensity]), and two levels due to imprecision (<400 participants, 95% CI inconsistent).  ^d^Downgraded two levels due to study limitations (mostly high risk of attrition bias, mostly high risk of performance and detection bias), two levels due to unexplained inconsistency (I2 = 96%), and one level due to indirectness (studies limited to certain participants [young and middle-aged adults], interventions [e.g., group setting, face-to-face delivery, moderate and high intensity, mindfulness-based training and combination] and comparators [no intervention, wait-list]). It was not downgraded for imprecision (95% CI wide and inconsistent) since the large inconsistency is likely to have also affected precision. | | | | | | |

**Supplementary table 3. PRISMA Statement**

| **Section/topic** | **#** | **Checklist item** | **Reported on page #** |
| --- | --- | --- | --- |
| **TITLE** | | |  |
| Title | 1 | Identify the report as a systematic review, meta-analysis, or both. | Both |
| **ABSTRACT** | | |  |
| Structured summary | 2 | Provide a structured summary including, as applicable: background; objectives; data sources; study eligibility criteria, participants, and interventions; study appraisal and synthesis methods; results; limitations; conclusions and implications of key findings; systematic review registration number. |  |
| **INTRODUCTION** | | |  |
| Rationale | 3 | Describe the rationale for the review in the context of what is already known. |  |
| Objectives | 4 | Provide an explicit statement of questions being addressed with reference to participants, interventions, comparisons, outcomes, and study design (PICOS). |  |
| METHODS | | |  |
| Protocol and registration | 5 | Indicate if a review protocol exists, if and where it can be accessed (e.g., Web address), and, if available, provide registration information including registration number. |  |
| Eligibility criteria | 6 | Specify study characteristics (e.g., PICOS, length of follow-up) and report characteristics (e.g., years considered, language, publication status) used as criteria for eligibility, giving rationale. |  |
| Information sources | 7 | Describe all information sources (e.g., databases with dates of coverage, contact with study authors to identify additional studies) in the search and date last searched. |  |
| Search | 8 | Present full electronic search strategy for at least one database, including any limits used, such that it could be repeated. |  |
| Study selection | 9 | State the process for selecting studies (i.e., screening, eligibility, included in systematic review, and, if applicable, included in the meta-analysis). |  |
| Data collection process | 10 | Describe method of data extraction from reports (e.g., piloted forms, independently, in duplicate) and any processes for obtaining and confirming data from investigators. |  |
| Data items | 11 | List and define all variables for which data were sought (e.g., PICOS, funding sources) and any assumptions and simplifications made. |  |
| Risk of bias in individual studies | 12 | Describe methods used for assessing risk of bias of individual studies (including specification of whether this was done at the study or outcome level), and how this information is to be used in any data synthesis. |  |
| Summary measures | 13 | State the principal summary measures (e.g., risk ratio, difference in means). |  |
| Synthesis of results | 14 | Describe the methods of handling data and combining results of studies, if done, including measures of consistency (e.g., I2) for each meta-analysis. |  |
| Risk of bias across studies | 15 | Specify any assessment of risk of bias that may affect the cumulative evidence (e.g., publication bias, selective reporting within studies). |  |
| Additional analyses | 16 | Describe methods of additional analyses (e.g., sensitivity or subgroup analyses, meta-regression), if done, indicating which were pre-specified. |  |
| **RESULTS** | | |  |
| Study selection | 17 | Give numbers of studies screened, assessed for eligibility, and included in the review, with reasons for exclusions at each stage, ideally with a flow diagram. |  |
| Study characteristics | 18 | For each study, present characteristics for which data were extracted (e.g., study size, PICOS, follow-up period) and provide the citations. |  |
| Risk of bias within studies | 19 | Present data on risk of bias of each study and, if available, any outcome level assessment (see item 12). |  |
| Results of individual studies | 20 | For all outcomes considered (benefits or harms), present, for each study: (a) simple summary data for each intervention group (b) effect estimates and confidence intervals, ideally with a forest plot. |  |
| Synthesis of results | 21 | Present results of each meta-analysis done, including confidence intervals and measures of consistency. |  |
| Risk of bias across studies | 22 | Present results of any assessment of risk of bias across studies (see Item 15). |  |
| Additional analysis | 23 | Give results of additional analyses, if done (e.g., sensitivity or subgroup analyses, meta-regression [see Item 16]). |  |
| **DISCUSSION** | | |  |
| Summary of evidence | 24 | Summarize the main findings including the strength of evidence for each main outcome; consider their relevance to key groups (e.g., healthcare providers, users, and policy makers). |  |
| Limitations | 25 | Discuss limitations at study and outcome level (e.g., risk of bias), and at review-level (e.g., incomplete retrieval of identified research, reporting bias). |  |
| Conclusions | 26 | Provide a general interpretation of the results in the context of other evidence, and implications for future research. |  |
| **FUNDING** | | |  |
| Funding | 27 | Describe sources of funding for the systematic review and other support (e.g., supply of data); role of funders for the systematic review. |  |

From: Moher D, Liberati A, Tetzlaff J, Altman DG, The PRISMA Group (2009). Preferred Reporting Items for Systematic Reviews and Meta-Analyses: The PRISMA Statement. PLoS Med 6(7): e1000097. doi:10.1371/journal. pmed1000097

For more information, visit: **www.prisma-statement.org**.

**Supplementary table 4. Characteristics of the included studies**

| Study | country | Publication  year | Sample （T/C) | Age | Intervention  (Training intensity) | Measurement  Time Point | Outcomes  Used |
| --- | --- | --- | --- | --- | --- | --- | --- |
| Fong et al.(26) | China | 2022 | 77(39/38) | 21-50 | mindful coloring (Practice for at least 5 days, each session: 20 min) | Baseline, end of treatment | ①② |
| Watanabe et al.(27) | Japan | 2019 | 80(40/40) | 21-55  (30.1±8.4) | Mindfulness-based stress reduction intervention (52 weeks; Four 30-min sessions per week) | Baseline, at weeks 13, 26 and 52 | ② |
| Xie et al.(28) | China | 2020 | 106(53/53) | 27.7 ± 7.7 | mindfulness-based intervention (8 weeks; once a week; each session: 2.5h) | Baseline, at 1 week, 1 month, and 3 months | ② |
| Yang et al.(29) | China | 2018 | 100(50/50) | 29.5±7.1 | mindfulness-based intervention (8 weeks; once a week) | Baseline, end of treatment(8 weceks) | ③④⑤ |
| Aghamohammadi et al.(30) | Iran | 2022 | 42(21/21) | 32.18 | mindfulness-based intervention (8 weeks; eight times a week; each session: 2 hours meeting and 20 minutes practice) | Baseline, end of treatment(8 weeks), and follow-up(3 months) | ① |
| Lin et al.(31) | China | 2019 | 90(44/46) | 31.50 | mindfulness-based intervention (8 weeks; eight times a week; each session: 90-120 minutes) | Baseline, end of treatment(8 weeks), and follow-up(3 months) | ① |
| Alexander et al.(32) | USA | 2015 | 40(20/20) | 46.38 ± 10.23 | Yoga (8 weeks) | Baseline, end of treatment(8 weeks) | ② |
| Duarte et al. (33) | Portugal | 2016 | 94(45/48) | 25-56  (41± 8.43) | mindfulness-based intervention (6 weeks;Weekly 2 h group sessions and daily home practice of at least 15 min) | Baseline, end of treatment(6 weeks) | ⑧ |
| Yıldırım et al.(34) | Turkey | 2022 | 104(52/52) | 27.55 ± 5.24/  29.11 ± 6.57 | Mindful breathing and music therapy(30min each) | Baseline, end of treatment | ⑥⑦ |
| Calisi (35) | USA | 2017 | 46(24/22) | 27 ~ 60 | Breathing exercises (8 weeks;10-20min twice a day) | Baseline, end of treatment(8 weeks) | ⑦ |
| Lin et al.(36) | China | 2018 | 58(27/31) | 33.12 | mindfulness-based intervention (8 weeks; 2h/week, personal practice 6 days/week, 20-30min/ day) | Baseline, end of treatment(8 weeks) | ① |
| Wei (37) | China | 2020 | 86(43/43) | 32.45 | Mindfulness-based stress reduction intervention (8 weeks; individual practice 6d/ week, 15-45min/ day, each session: 2h) | Baseline, end of treatment(8 weeks), follow-up(3 months) and follow-up(6 months) | ①② |
| Wang et al.(38) | China | 2018 | 75((38/37) | 32.6 ±4.2/  33.2 ±4.0 | mindfulness-based intervention (8 weeks; 2 hours every Monday, once a week for 10 sessions) | Baseline, end of treatment(3 months) | ⑨ |
| Wang et al.(39) | China | 2017 | 66(33/33) | 23~42  (28.20±5.80) | mindfulness-based intervention (8 weeks; Once a week, 2 hours each time) | Baseline, end of treatment(8 weeks) | ⑩ |
| Yang et al.(40) | China | 2022 | 101(50/51) | 38.36±6.47/  37.84±7.60 | mindfulness-based intervention (8 weeks; 2.5 hours per week, and a half-day stop speech retreat) | Baseline, end of treatment(8 weeks), and follow-up(3 months) | ② |
| T: stands for intervention group, C: stands for control group; (1) Perceived stress Scale (PSS/PSS-10/CPSS); (2) Maslach Burnout Inventory (MBI/MBI-HSS-MP/MBI-HSS/MBI-GS); (3) Self-rating anxiety Scale (SAS); (4) Self-rating depression scale (SDS); (5) Nursing Stress Scale (6) Job Stress Scale (7) State Anxiety Inventory (STAI-I) (8) Quality of Professional Life Scale, Fifth edition (ProQOL-5) (9) Symptom Checklist 90 (SCL-90) (10) Nurse Stress Scale | | | | | | | |

**Supplementary Table 5. Risk of bias judgments for randomized controlled trials**

| **Study** | **Random sequence generation** | **Allocation concealment** | **Blinding of participants and personnel** | **Blinding of outcome assessment** | **Incomplete outcome data** | **Selective reporting** | **Other bias** | **Quality grade** |
| --- | --- | --- | --- | --- | --- | --- | --- | --- |
| Fong et al.(26) | L | L | U | L | L | L | L | B |
| Watanabe et al.(27) | L | L | H | L | L | L | L | B |
| Xie et al.(28) | L | L | H | L | L | L | L | B |
| Yang et al.(29) | L | L | H | U | L | L | L | B |
| Aghamohammadi et al.(30) | U | U | H | L | L | L | L | B |
| Lin et al.(31) | L | L | U | U | L | L | L | B |
| Alexander et al.(32) | L | L | H | L | L | L | U | B |
| Duarte et al. (33) | L | L | U | L | L | L | L | B |
| Yıldırım et al.(34) | L | L | U | L | L | L | L | B |
| Calisi (35) | L | L | H | L | L | L | L | B |
| Lin et al.(36) | L | L | H | U | L | L | L | B |
| Wei (37) | L | U | U | L | L | L | L | B |
| Wang et al.(38) | L | U | H | L | L | L | U | B |
| Wang et al.(39) | L | U | H | L | L | L | U | B |
| Yang et al.(40) | L | L | H | L | L | L | L | B |
| L, low risk ; H, high risk; U, unclear risk. | | | | | | | |  |

## Supplementary Figures

**
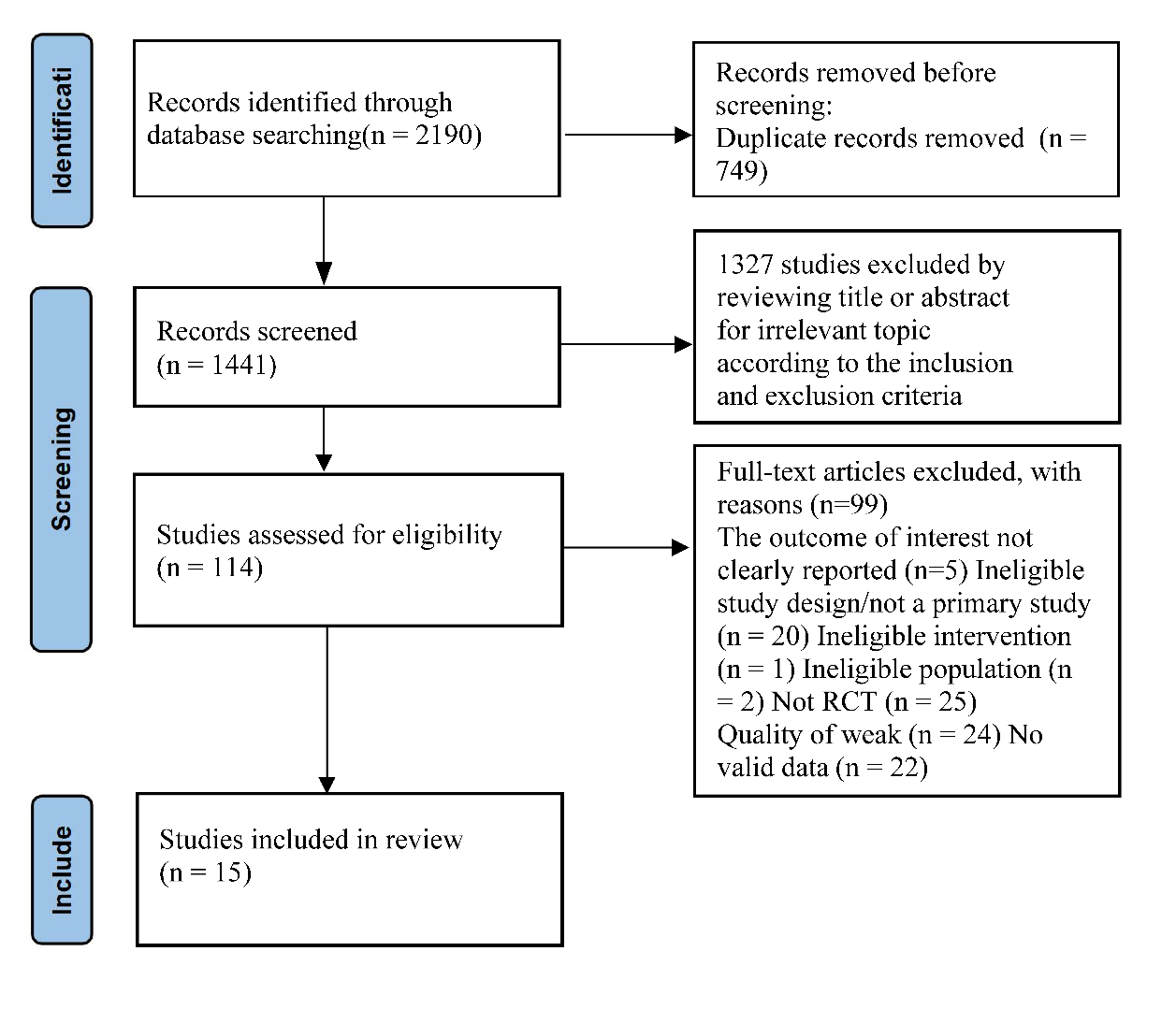
**

**Supplementary Figure 1. PRISMA diagram summarizing the records retrieval and workflow.**

**
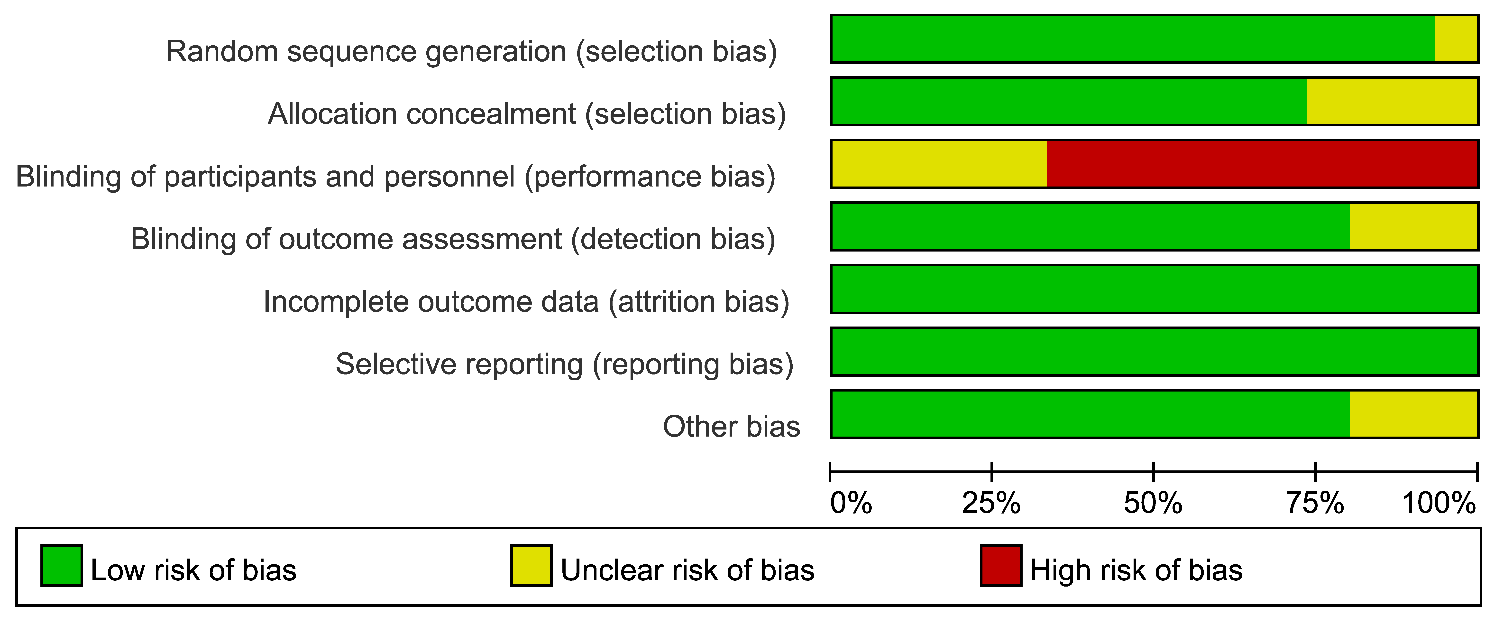
**

**Supplementary Figure 2.** **Risk of bias graph of RCTs**

**
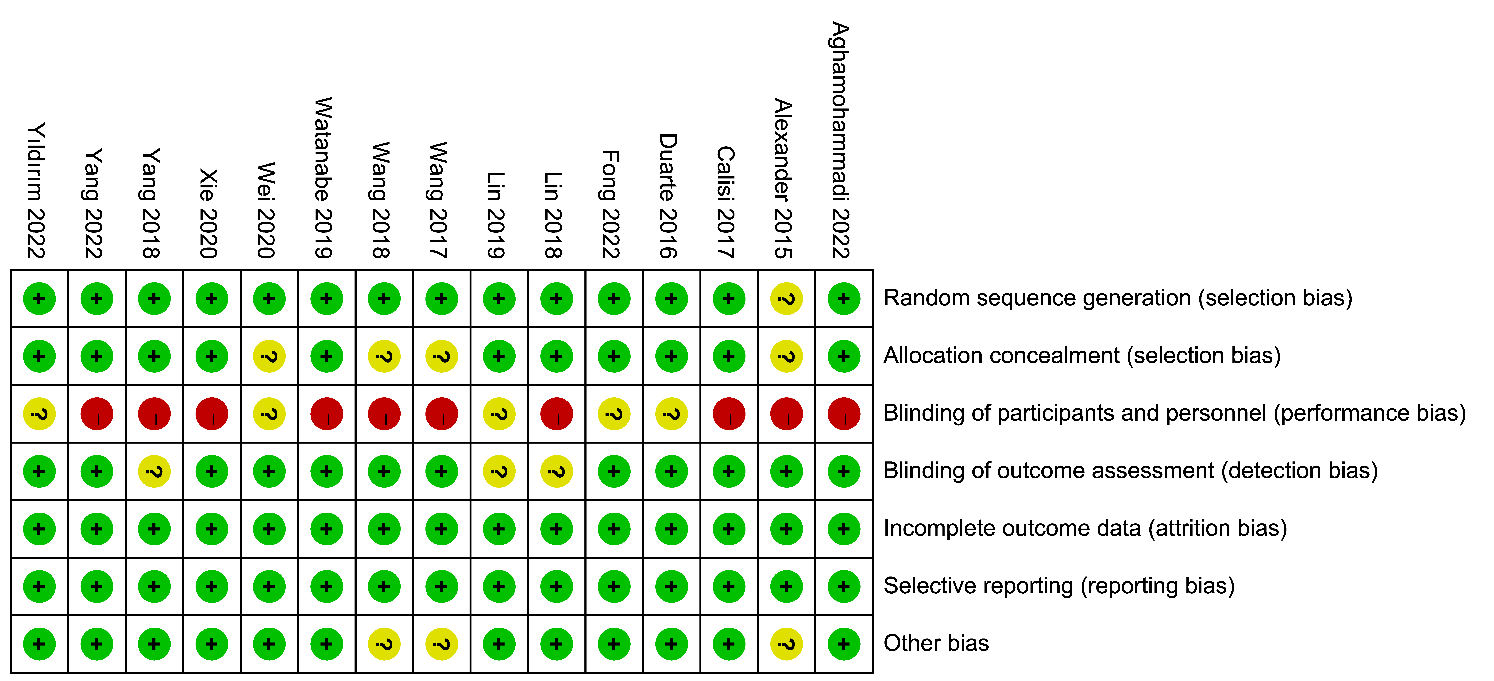
**

**Supplementary Figure 2.Risk of bias summary**

**
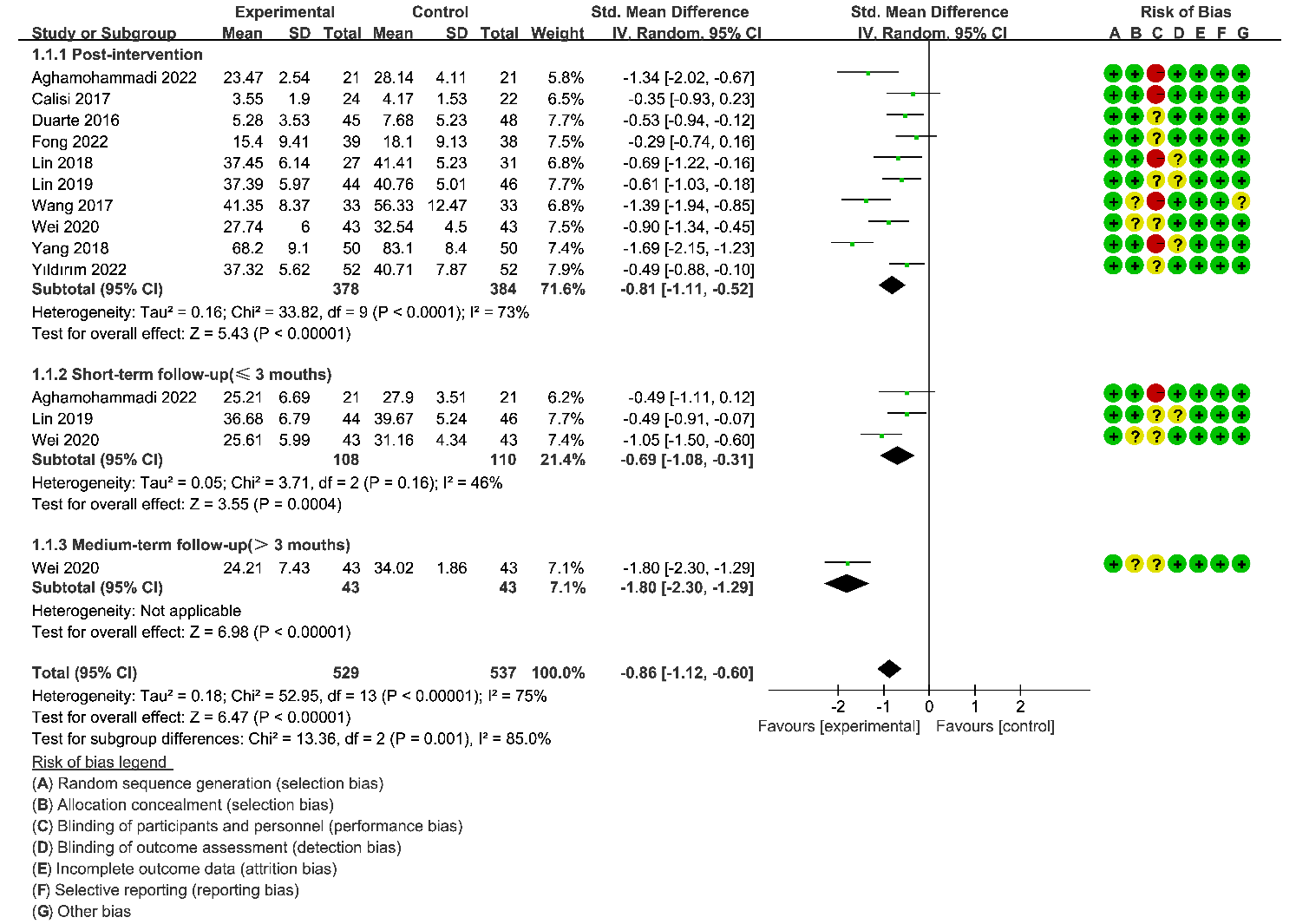
**

### Supplementary Figures 3.The forest plot of (perceived) stress at different time points

### Supplementary Figures 4. The forest plot of burnout at different time points


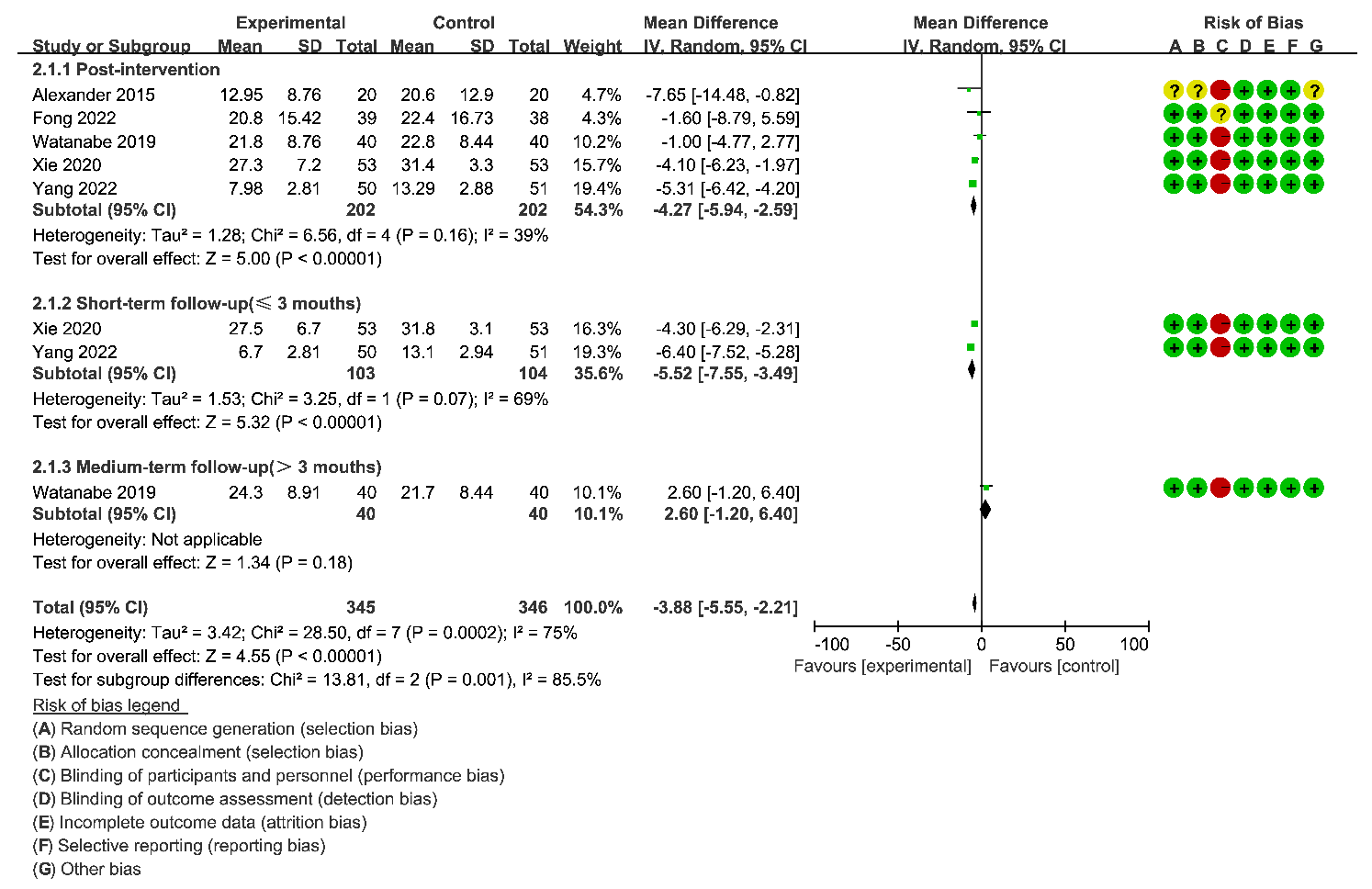


1. **Emotional exhaustion**

**
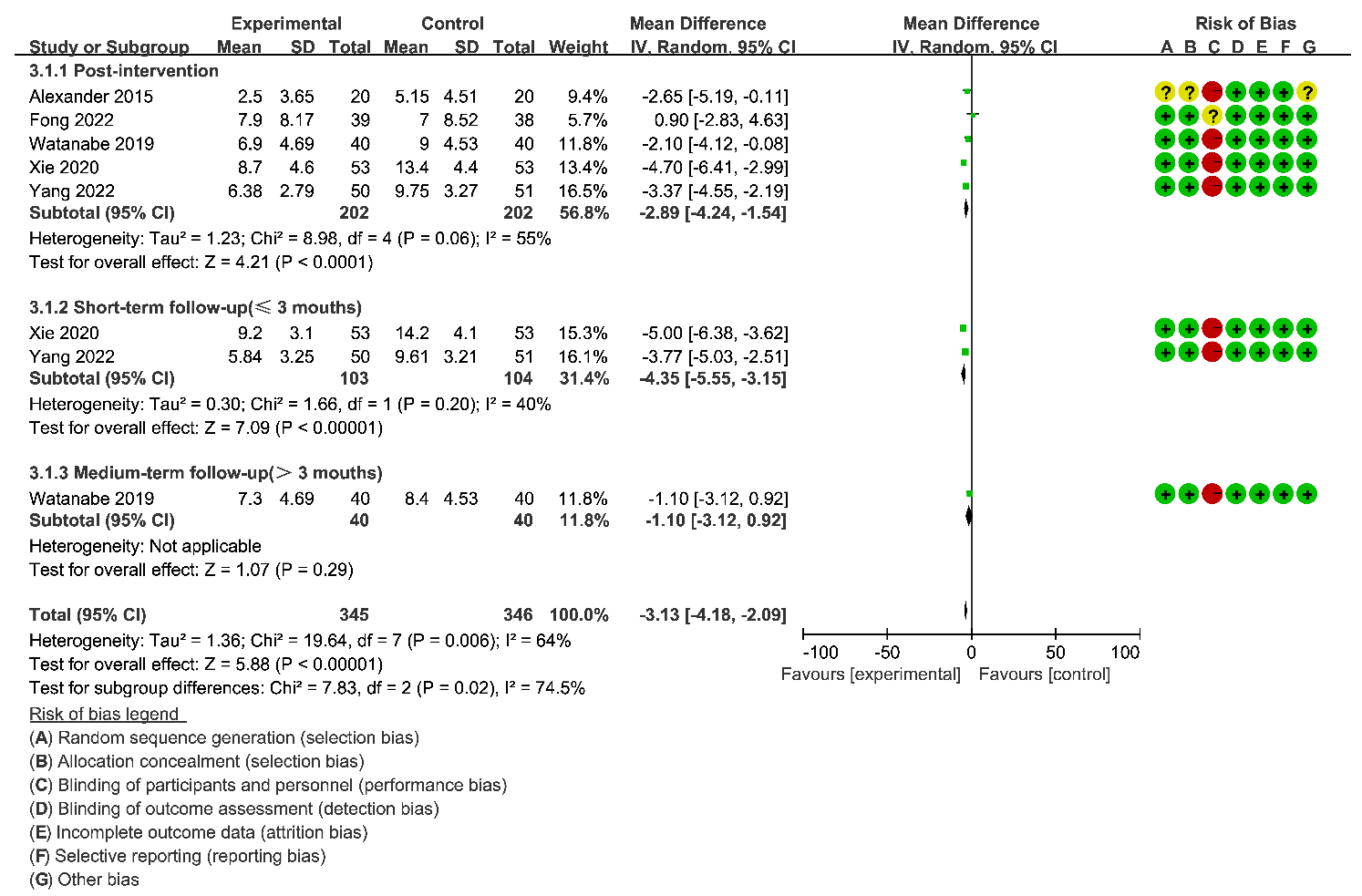
**

1. **Depersonalization**

**
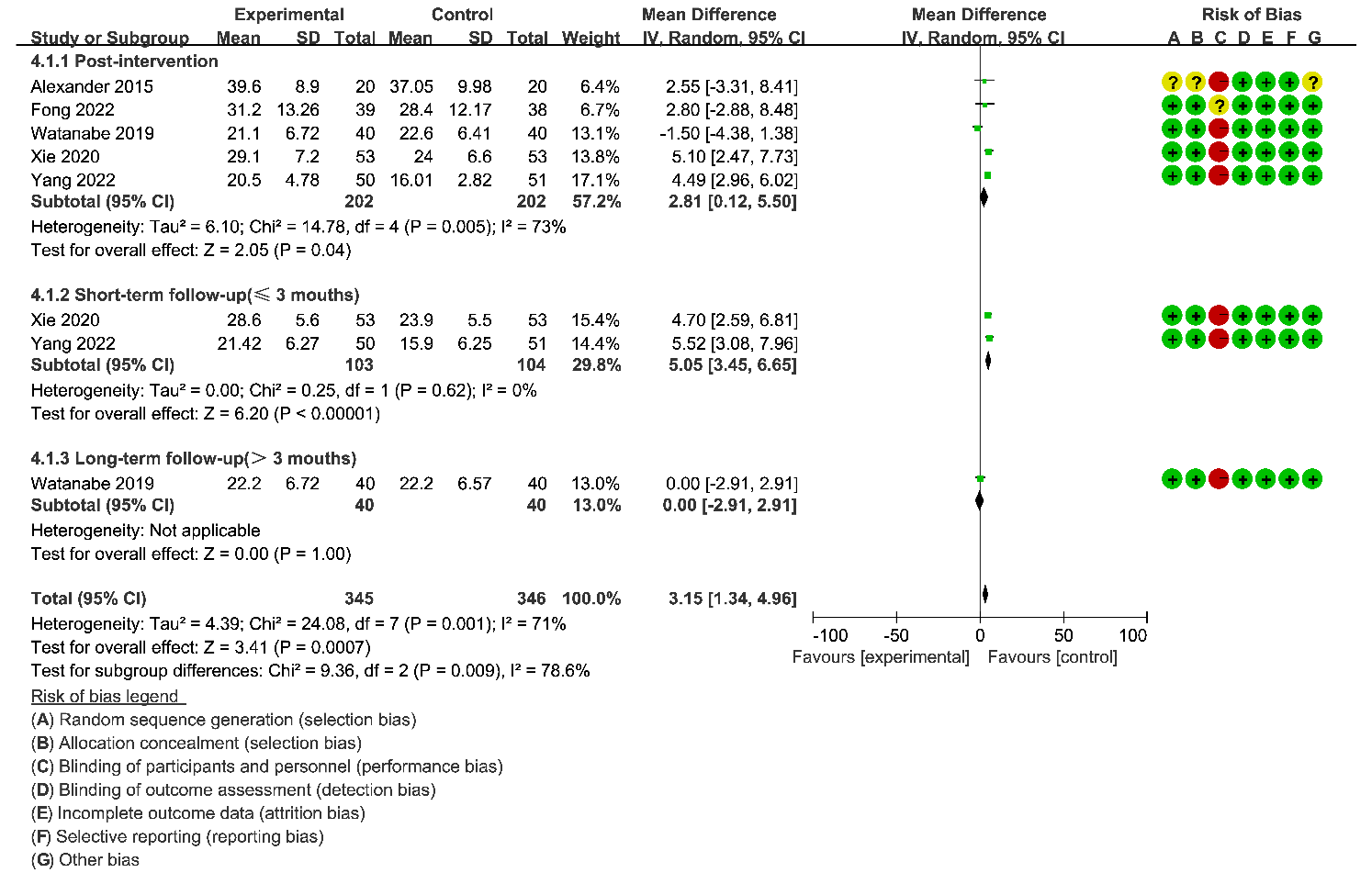
**

1. **Personal accomplishment**

**
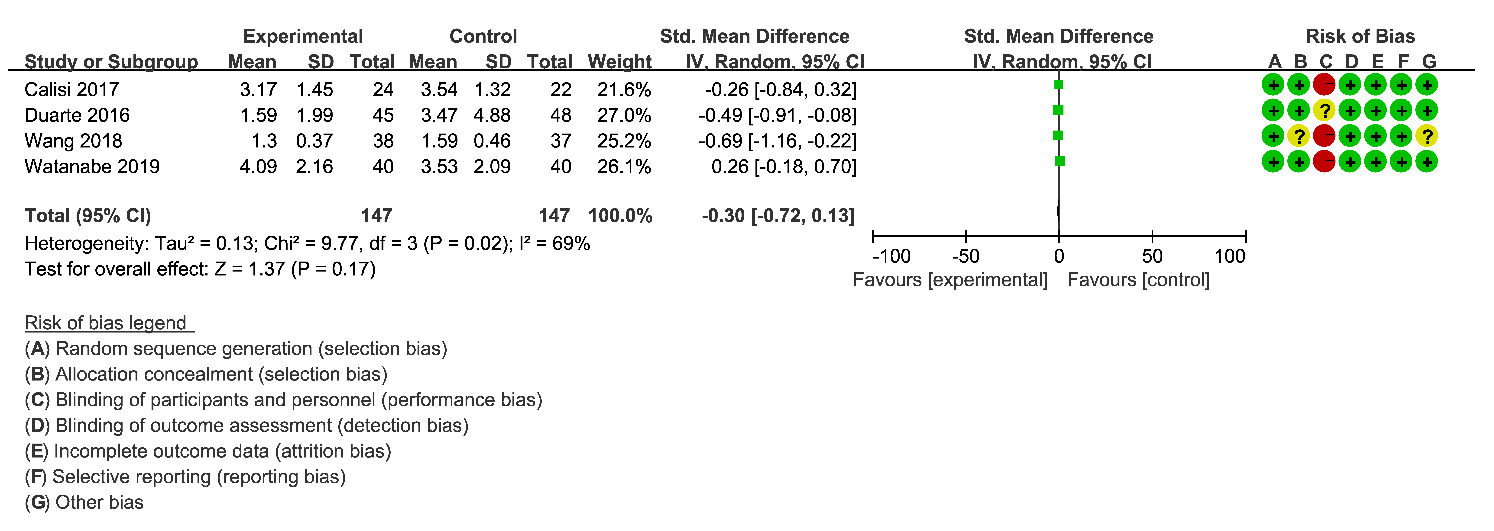
**

**Supplementary Figures 5.The forest plot of anxiety at different time points**

**
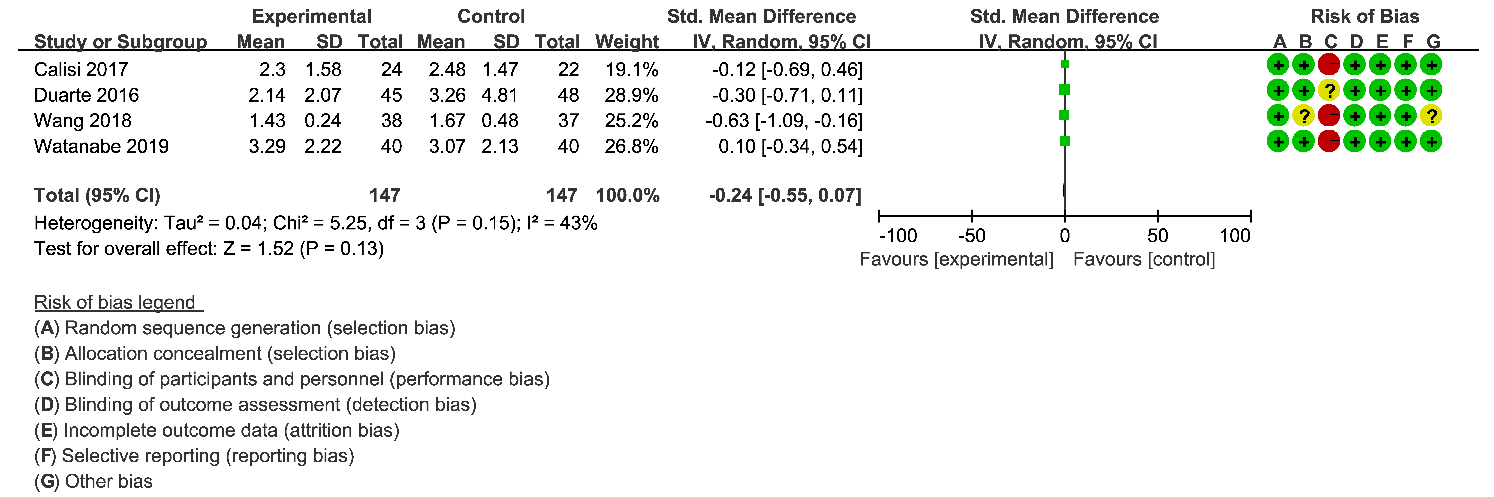
**

### Supplementary Figures 6.The forest plot of depression at different time points

**Supplementary Figures 7.The forest** **plot of subgroup analysis**


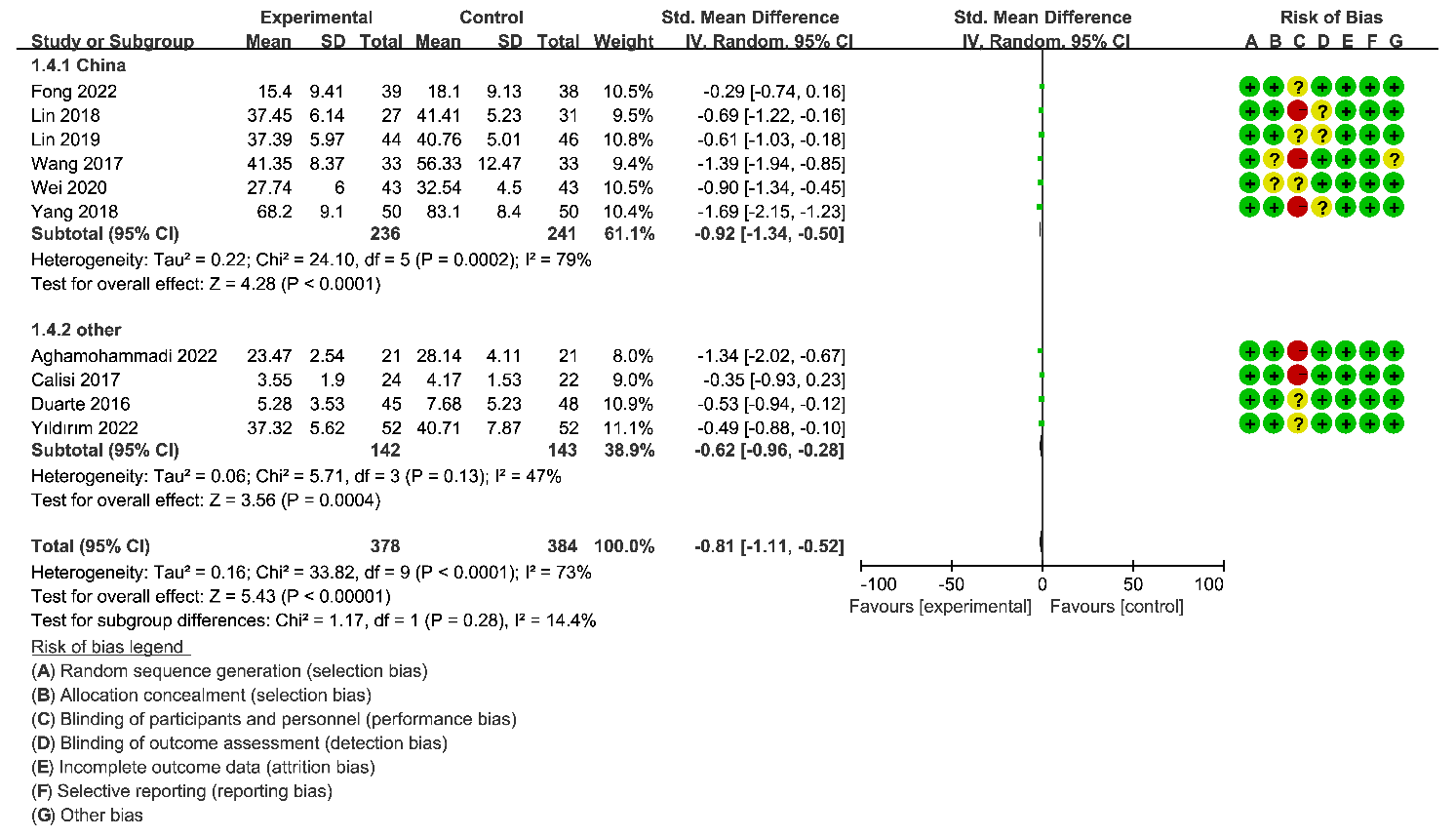


1. **Subgroup analysis regarding the country of study**

**
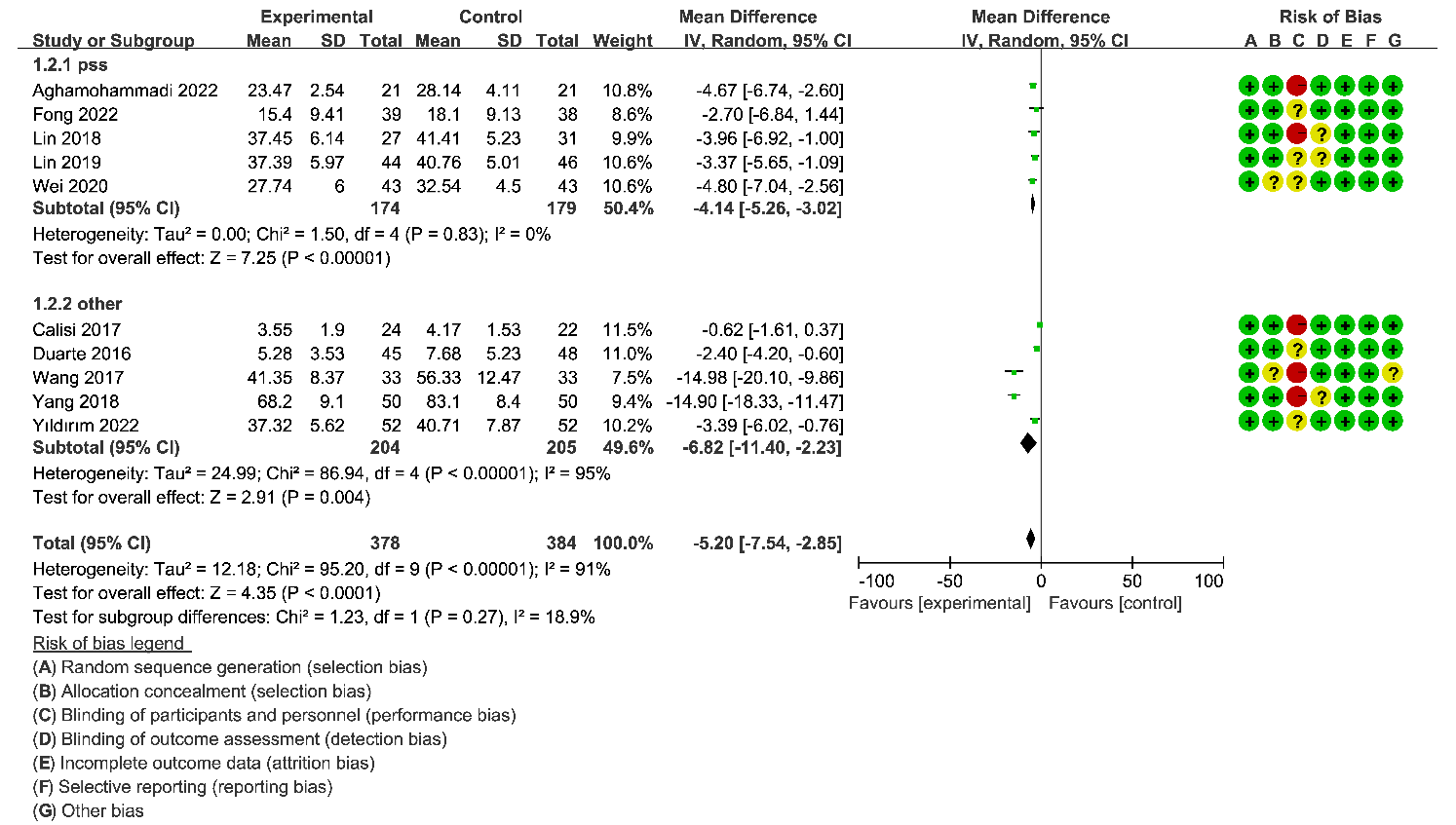
**

1. **Subgroup analysis** **regarding the outcome measures of study**

**
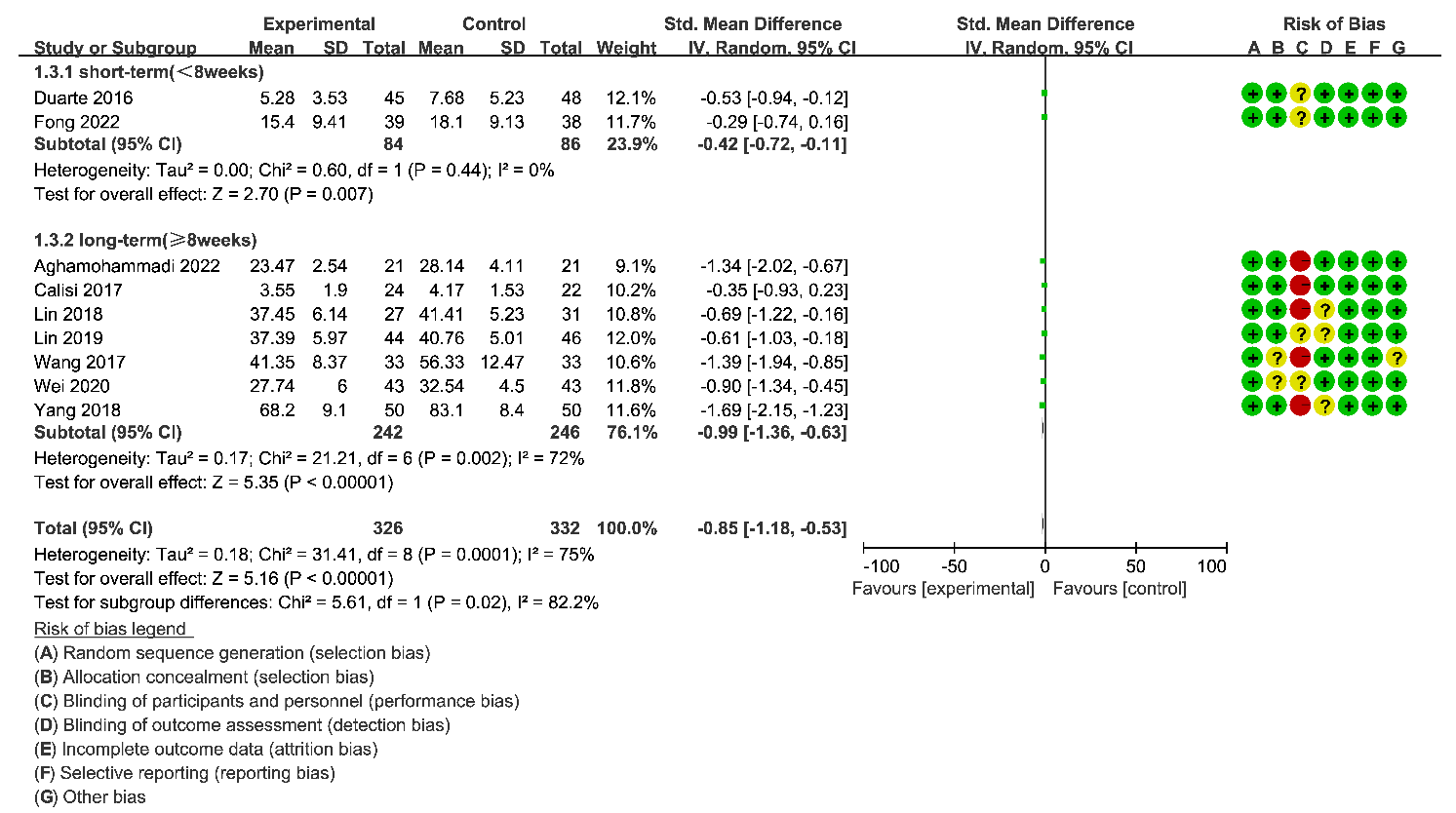
**

1. **Subgroup analysis regarding the intervention duration of study**

**Supplementary Figures 8. sensitivity analysis.**

**
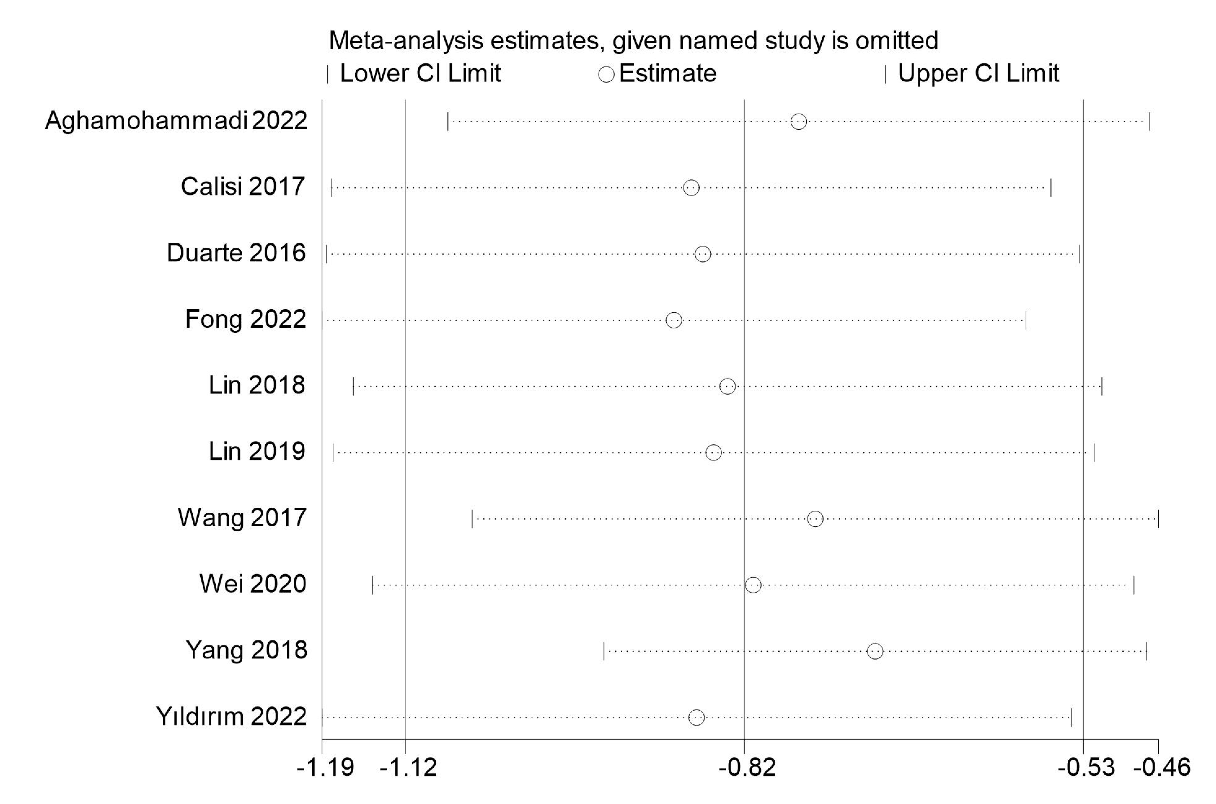
**

1. **Pressure sensitivity analysis**

**
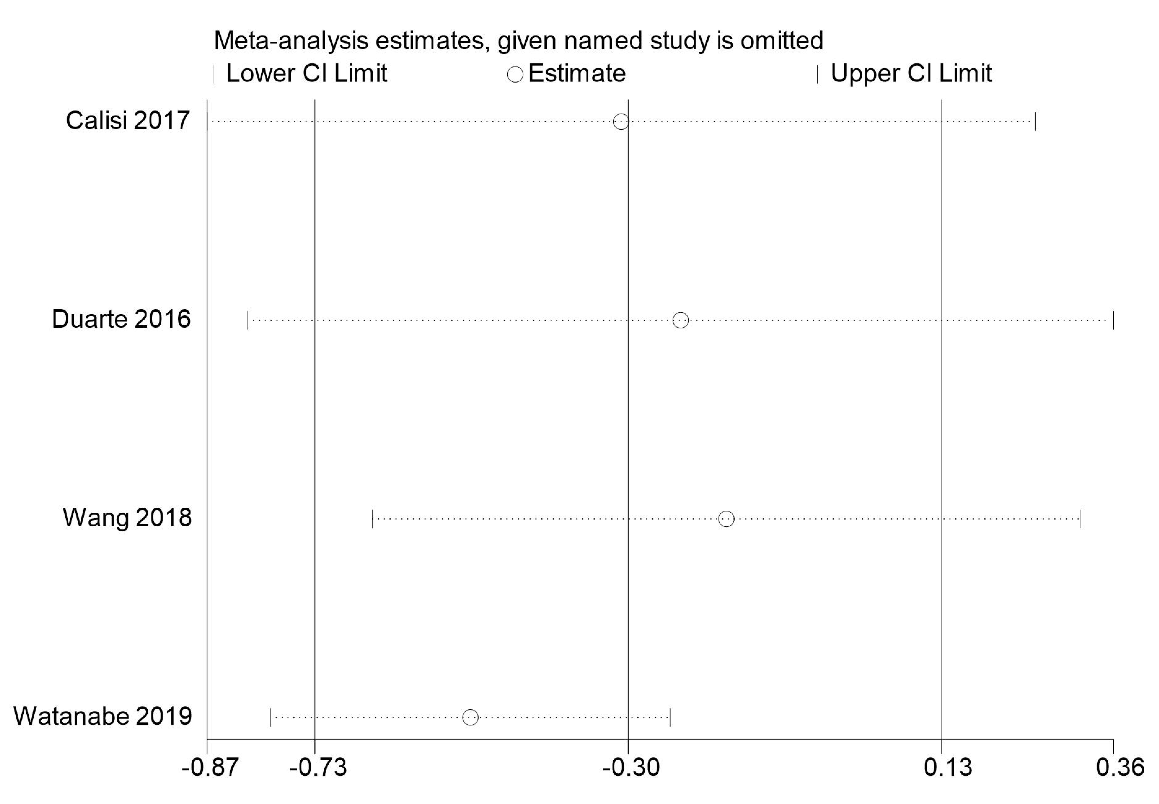
**

1. **Anxiety sensitivity analysis**

**
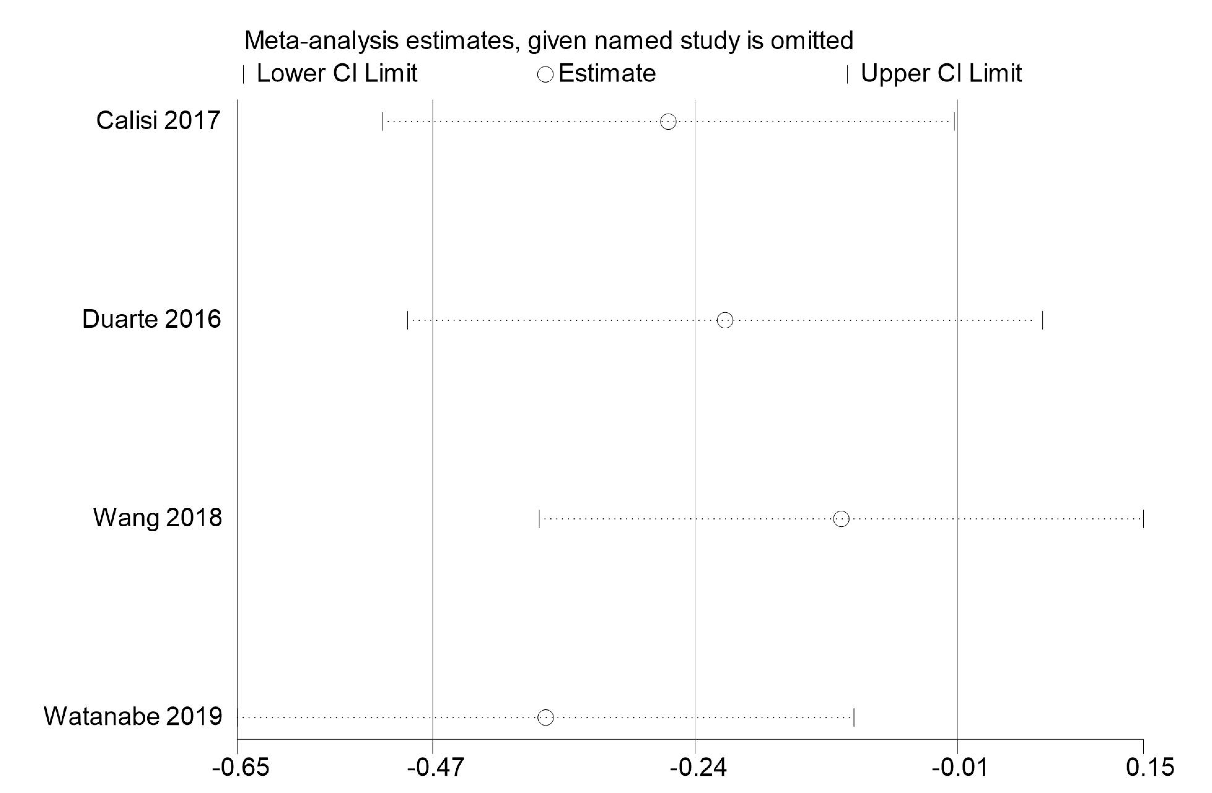
**

1. **Depression sensitivity analysis**
